# Xanthomatous Hypophysitis: A Case Report and Comprehensive Literature Review

**DOI:** 10.3389/fendo.2021.735655

**Published:** 2021-10-01

**Authors:** Jianyu Zhu, Zhicheng Wang, Wenze Wang, Jinghua Fan, Yi Zhang, Xiaoxu Li, Jie Liu, Shenzhong Jiang, Kan Deng, Lian Duan, Yong Yao, Huijuan Zhu

**Affiliations:** ^1^ Department of Neurosurgery, Peking Union Medical College Hospital, Chinese Academy of Medical Science and Peking Union Medical College, Beijing, China; ^2^ Department of Neurosurgery, The First Affiliated Hospital of Fujian Medical University, Fuzhou, China; ^3^ Department of Pathology, Peking Union Medical College Hospital, Chinese Academy of Medical Science and Peking Union Medical College, Beijing, China; ^4^ Key Laboratory of Endocrinology of National Health Commission, Department of Endocrinology, Peking Union Medical College Hospital, Chinese Academy of Medical Science and Peking Union Medical College, Beijing, China

**Keywords:** xanthomatous hypophysitis, clinical characteristics, pathological examination, surgery, recurrence

## Abstract

**Purpose:**

Xanthomatous hypophysitis (XHP) is an extremely rare form of primary hypophysitis for which there is a lack of clinical experience. A comprehensive understanding of its clinical characteristics, diagnosis and treatment is needed.

**Methods:**

Here, we report a case study and conduct a systematic review of XHP. Thirty-six cases were included, and their clinical manifestations, endocrine assessment, imaging features, treatment and follow-up data were collected and analyzed.

**Results:**

The mean age at diagnosis was 39.1 years, and females were predominant (75.0%). The most common symptom was headache (68.6%), and 66.7% of female patients presented menstrual disorders. The most common pituitary dysfunction was growth hormone (GH) deficiency. More than half of patients exhibited central diabetes insipidus (CDI). The majority of patients had an imaging presentation of a cystic lesion with peripheral enhancement. Pituitary stalk thickening was observed in half of the patients. Total lesion resection was achieved in 57.1% of cases. The recurrence rate after partial resection and biopsy was significantly higher than that after total lesion resection (57.1% *vs.* 0.0%, P = 0.0147). The most common pituitary hormone abnormalities to resolve after surgery were hyperprolactinemia (100.0%) and GH deficiency (91.7%). The typical pathological feature was inflammatory infiltration of foamy histiocytes, which showed positivity for CD68.

**Conclusion:**

Diagnosis of XHP is difficult when relying on clinical symptoms and imaging features. Therefore, surgical histopathology is necessary. Based on the available evidence, total lesion resection is recommended for treatment. However, the long-term prognosis for this rare disease remains unclear.

## Introduction

Hypophysitis is a rare disease characterized by lymphocytic infiltration of the pituitary gland, which can be divided into primary and secondary types (1, 2). Primary hypophysitis is the most common type, including several heterogeneous forms (1, 2). XHP is thought to be the rarest subtype of primary hypophysitis, and there is a lack of clinical experience with this disease (2). In this study, we report a case of XHP in our center and review all of the reported cases in previous literature. The objective of this study is to summarize the clinical characteristics and to bring an up-to-date understanding of XHP to provide evidence for the diagnosis, differential diagnosis and treatment of this rare disease.

## Methods

**Figure 1:**
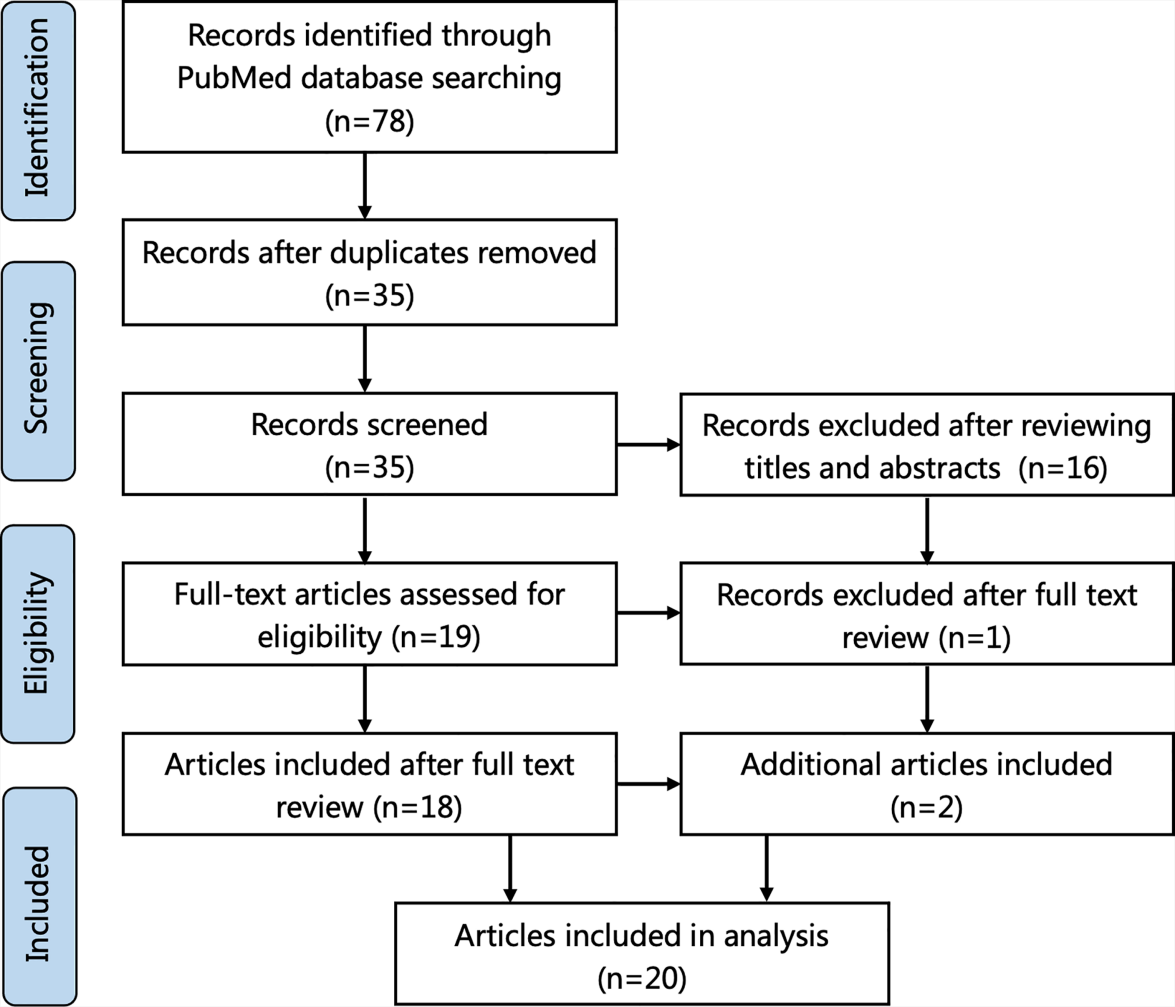
Flowchart of literature selection using the PRISMA guidelines.

A review of the literature was performed in accordance with PRISMA guidelines. The terms “xanthomatous hypophysitis” and “xanthomatous AND hypophysitis” were searched using the online database PubMed until May 2021. Cases whose diagnosis of XHP was confirmed by histological examination were included. Review articles without new cases were excluded. The relevant adequate cases mentioned in the references of these articles were also considered. The search flowchart is shown in **Figure 1**. lf total of 35 applicable cases reported in 20 articles were finally included.

Information on clinical symptoms, laboratory results, radiological features, surgery and postoperative conditions was extracted from these articles. Continuous data are expressed as the means ± standard deviations; categorical variables are summarized as frequencies and percentages and were analyzed using the χ^2^ test or Fisher’s exact test. The data were analyzed using GraphPad Prism, version 8 (GraphPad Software, San Diego, California, USA).

## Results

### Case Report

**Figure 2:**
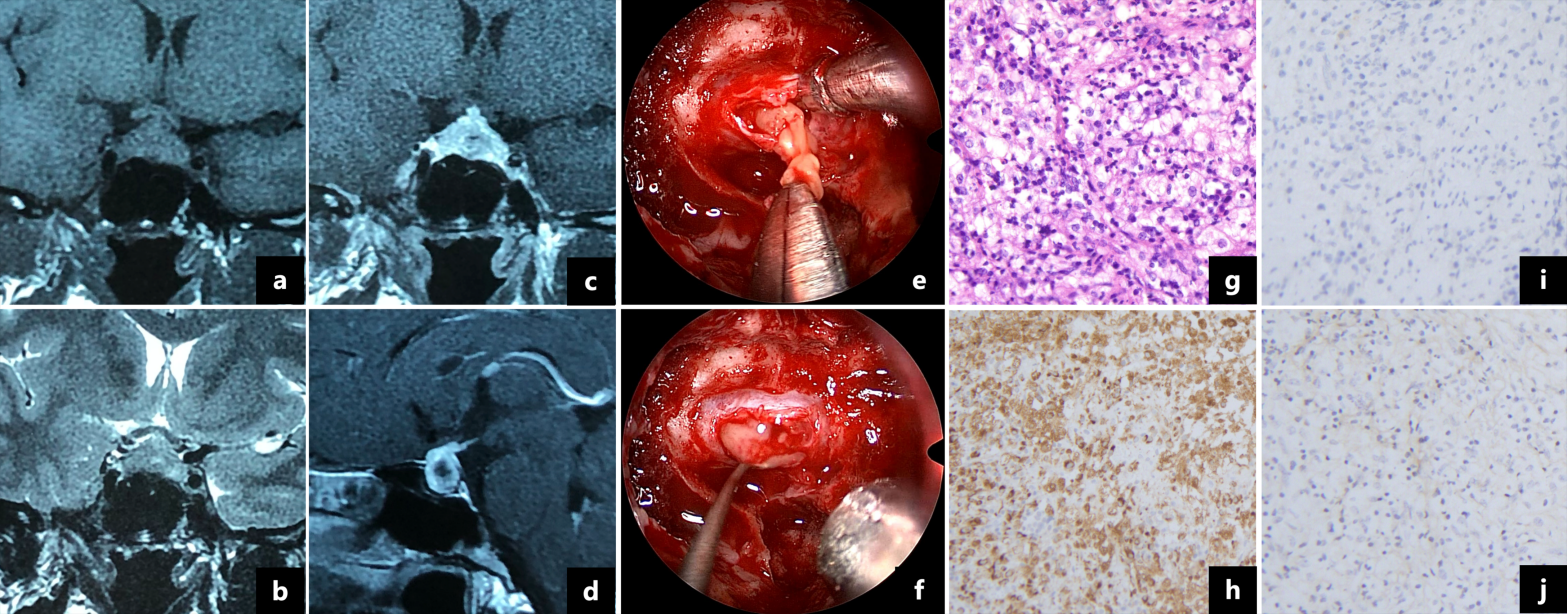
Magnetic resonance imaging showed a 1.7×1.2 cm cystic lesion with a thickened pituitary stalk and mainly peripheral enhancement **(A–D)**. Intraoperative pictures revealed a soft yellow lesion **(E)** with pus-like fluid **(F)**. Pathologic examination showed that the pituitary gland was infiltrated by foamy histocytes and lymphocytes (**G**, ×200). The foamy cells were immunopositive for CD68 (**H**, ×200) and immunonegative for CD1a (**I**, ×200) and S-100 protein (**J**, ×200).

A 36-year-old woman presented with a 5-month history of headaches, polyuria and polydipsia and a 2-month history of amenorrhea. Her daily water intake was 3500-5000 ml. A water deprivation test indicated central diabetes insipidus (CDI). The results of the pituitary hormone test are shown in **Table 1**. Her anti-peroxidase antibody (>600 IU/mL, normal range<34 IU/mL) and anti-thyroglobulin antibody (730 IU/mL, normal range<115 IU/mL) were high. Thyroid gland ultrasonography showed a normal volume with diffuse heterogeneous background echotexture of both thyroid glands. A visual field test showed partial bilateral temporal defects. A pituitary magnetic resonance imaging (MRI) scan revealed a 1.7×1.2 cm cystic lesion occupying the sella turcica with mainly peripheral enhancement (**Figures 2A–D**). Desmopressin (0.05 mg) was administered orally three times per day for DI as hormone replacement therapy. Endoscopic transsphenoidal surgery was performed and displayed a soft yellowish lesion with pus-like discharge (**Figures 2E, F**). The lesion was totally removed. The pathologic examination showed inflammatory infiltration of normal pituicytes by foamy histiocytes and lymphocytes, with fibrosis, local cysts and necrosis. Immunohistochemistry of the foamy histiocytes indicated strong staining for CD68 (**Figures 2G–J**).

**Table 1:** The results of the pituitary hormone test in our case.

Test projects	Normal range	Before surgery	After surgery (1 months)	After surgery (6 months)*
GH (ng/mL)	<2.0	3.1	1.5	1.8
IGF-1 (ng/mL)	115-320	352	288	209
Morning cortisol (μg/dL)	4.0-22.3	7.4	7.0	10.7
ACTH (pg/mL)	0-46	19.3	29.6	15.7
TSH (μIU/mL)	0.38-4.34	3.015	14.827	2.624
Free T3 (pg/mL)	1.80-4.10	2.61	3.49	2.58
Free T4 (ng/dL)	0.81-1.89	0.78	1.19	1.13
LH (IU/L)	2.12-10.89	3.23	3.35	6.27
FSH (IU/L)	<10	6.79	5.80	8.45
E2 (pg/mL)	22-115	<15	74	25
P (ng/ml)	0.38-2.28	0.27	6.18	0.49
T (ng/mL)	0.10-0.75	0.31	<0.1	0.20
PRL (ng/mL)	<30.0	67.1	21.0	29.2

*The hormones were measured on the third day of the patient’s menstrual cycle.

GH, growth hormone; IGF-1, insulin-like growth factor-1; ACTH, adrenocorticotropic hormone; TSH, thyroid-stimulating hormone; T3, triiodothyronine; T4, thyronine; LH, luteinizing hormone; FSH, follicle-stimulating hormone; E2, estradiol; P, progesterone; T, testosterone; PRL, prolactin.

The patient was discharged with no surgical complications. Postoperatively, her headaches and visual field defects resolved. The dose of desmopressin decreased during follow-up, and levothyroxine was administered because of autoimmune thyroiditis. The patient’s menstruation resumed three months after surgery. At the 6-month follow-up, desmopressin was discontinued, and the pituitary hormones were within the normal range (**Table 1**).

### Literature Review

**Figure 3:**
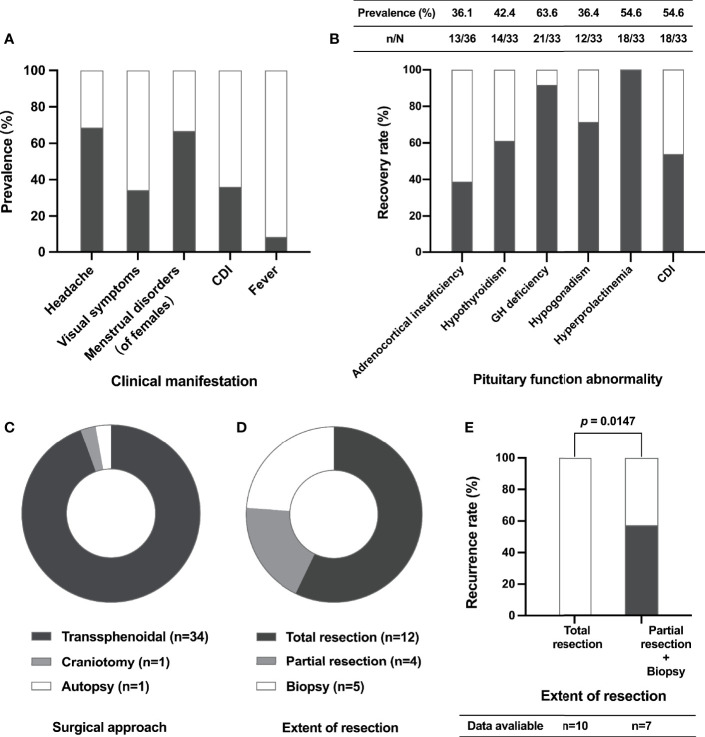
Clinical characteristics, surgical aspects and recurrence of XHP cases. The prevalence of different clinical manifestations of XHP patients **(A)**; the prevalence of pituitary function abnormalities before surgery (table) and the recovery rate of pituitary function after surgery (histogram) **(B)**; the composition of different surgical approaches **(C)**; the composition of different extents of lesion resection **(D)**; the relationship between recurrence and extent of resection **(E)**. CDI, central diabetes insipidus; GH, growth hormone.

There were 36 cases of XHP, including 35 in previous literature (3–22) and one in our center (summarized in **Table 2**). The mean age at diagnosis was 39.1 years (range 12-72), and 27 (75.0%) patients were female. The most common symptoms were headache (68.6%), visual impairment (34.3%) and diabetes insipidus (DI) (36.1%). A total of 66.7% of female patients presented menstrual disorders, and three (8.3%) patients presented fever (**Figure 3A**). In addition, apoplectic events occurred in two patients (10, 13). Interestingly, five patients had autoimmune disease, including two who had autoimmune thyroiditis (12), one who had rheumatoid arthritis (13), one who had ulcerative colitis (9) and one who had both rheumatoid arthritis and Sjogren’s syndrome (16).

**Table 2:** Review of 36 cases of xanthomatous hypophysitis.

Case	Reference	Sex/Age (years)	MRI presentation	Gross appearance	Pathologic findings	IHC	Treatment	Recurrence	Follow-up (months)
1	Folkerth et al., 1998 (3)	F/30	Cystic sellar mass	Fibrous capsule with “purulent” material	Foamy histiocytes and lymphocytes	CD68(+), CD1a(-), S-100(-)	TSS	N	8
2		F/30	Sellar and suprasellar mass with ring enhancement	Soft lesion with white material	Foamy histiocytes and lymphocytes	CD68(+), CD1a(-), S-100(-)	TSS	N	9
3		F/12	Sellar mass with peripheral enhancement, PST	Cystic lesion containing pus-like fluid	Foamy histiocytes and lymphocytes	CD68(+), CD1a(-), S-100(-)	TSS+HRT	NA	NA
4	Deodhare et al., 1999 (4)	F/43	Intrasellar and suprasellar lesion, PST	Cystic lesion containing pus-like fluid	Foamy histiocytes, lymphocytes, and fibrosis	CD68(+), CD1a(-), S-100(-)	TSS+HRT	N	2
5	Cheung et al., 2001 (5)	F/32	Cystic lesion, PST	Cyst with fibrous wall containing orange material	Foamy histiocytes, lymphocytes, hemosiderin and fibrosis	NA	Surgery	N	24
6	Tashiro et al., 2002 (6)	F/43	NA	NA	Foamy histiocytes and lymphocytes	S-100(-)	Autopsy	—	—
7		F/72	NA	NA	Foamy histiocytes and lymphocytes	S-100(-)	Surgery	NA	NA
8	Burt et al., 2003 (7)	M/29	Pituitary mass extending into suprasellar cistern with peripheral enhancement	Tense cystic lesion containing yellow fluid	Foamy histiocytes, lymphocytes, cholesterol clefts, and multinucleated giant cells	CD68(+), CD1a(-)	TSS+HRT	N	18
9		M/26	Lobulated intrasellar and suprasellar mass	Greenish-tinged partially cystic thick-walled lesion containing thick brown fluid	Foamy histiocytes, lymphocytes, hemosiderin, cholesterol clefts, and fibrosis	CD68(+), CD1a(-)	TCS+HRT	N	8
10	Gutenberg et al., 2006 (8)	3F/ Average age: 29.0 ± 2.7	Mild enhancing well-defined intrasellar mass, PST (1/3)	NA	NA		Surgery+HRT	NA	NA
11									
12									
13		M/41							
14	Aste et al., 2010 (9)	F/31	Solid lesion with necrotic core and hemorrhagic spot, PST	Cystic lesion containing pus-like fluid	Foamy histiocytes, lymphocytes, cholesterol clefts, multinucleated giant cells	CD68(+)	TSS+HRT	N	8
15	Haas et al., 2012 (10)	M/57	Cystic lesion with peripheral ring enhancement	Emergence of a thick yellow fluid	Foamy histiocytes and lymphocytes	CD68(+)	TSS+HRT	N	6
16	Niyazoglu et al., 2012 (11)	F/39	Sella lesion with a necrotic/cystic core	NA	Foamy histiocytes	CD68(+), CD1a(-), S-100(-)	TSS+HRT	N	3
17	Joung et al., 2013 (12)	F/36	Cystic lesion with peripheral enhancement, PST	Friable, encapsulated mass with moderate vascularity	Foamy histiocytes	CD68(+), CD1a(-), S-100(-)	TSS+HRT+High-dose glucocorticoid therapy	Y	10
18	Hanna et al., 2015 (14)	F/69	Pituitary mass with peripheral enhancement, PST	Red-tan colored tissue; fibrous and with a firm rubbery texture, consisting of friable red tissue	Histiocytes and lymphoid infiltrate	CD68(+), CD1a(-), S-100(-)	TSS+HRT→TSS+HRT+RT	Y	3
19	Tang et al., 2015 (15)	F/33	Solid sellar and suprasellar mass surrounding the left cavernous, PST	Solid lesion with rich blood supply and tough texture	Foamy histiocytes, lymphoplasmacytes and fibrosis	CD68(+), S-100(+), CD1a(-)	TSS	NA	NA
20	Gopal Kothandapani et al., 2015 (13)	F/14	Pituitary mass with heterogeneous enhancement and central necrosis	NA	Necrosis, cholesterol clefts, fibrosis and hemosiderin	NA	TSS+HRT	N	6
21		F/21	Pituitary lesion with suprasellar extension	NA	Necrosis, cholesterol clefts, fibrosis, multinucleated giant cells and hemosiderin	CD68(+)	TSS+HRT	N	6
22		M/67	Cystic pituitary mass	NA	NA	NA	TSS→TSS+High-dose steroids and methotrexate	Y	12
23	Oishi et al., 2016 (16)	F/72	Multicystic lesion extending toward the hypothalamus, PST	Soft yellowish lesion along with the pituitary stalk	Infiltrating foamy cells with lymphocytes	CD68(+), CD1a(-), S-100(-)	TSS+HRT	NA	NA
24	Duan et al., 2017 (17)	5F, 2M/ Average age: 43.3 ± 18.3	NA	NA	Foamy macrophages and multinucleated giant cells	NA	TSS	NA	NA
25			Suprasellar extension	Cystic component with thick yellowish fluid					
26			Suprasellar extension, compression of the optic chiasm and extension into the left cavernous sinus	Cystic component					
27			Suprasellar extension, PST	Cystic component with a thick yellowish fluid					
28			Suprasellar extension, compression of the optic chiasm	Mucinous and speck-like					
29			Suprasellar extension	Cystic component with yellowish mucous material					
30			Suprasellar extension, compression of the optic chiasm	Hemorrhagic cystic component					
31	Lin et al., 2017 (18)	F/41	Sellar lesion with peripheral ring enhancement, PST	Friable yellow hypovascular mass	Foamy histiocytes and lymphocytes	CD68(+)	TSS	N	15
32	Singh et al., 2018 (19)	M/18	Heterogenous enhancing mass, PST	Yellow mass with irregular necrotic tissue and thick, yellow fluid pus	Foamy macrophages	CD68(+)	TSS+HRT	NA	NA
33	Kini et al., 2019 (21)	F/55	Sellar mass with suprasellar extension	NA	Foamy histiocytes and lymphoplasmacytic infiltrate with interspersed fibrosis and hyalinization	CD68(+)	TSS	NA	NA
34	Imga et al., 2019 (20)	M/27	Sellar mass with heterogenous enhancement, PST	NA	NA	NA	TSS	N	28
35	Mathkour et al., 2020 (22)	F/45	Cystic lesion with suprasellar extension	Mass with thick yellow colloidal material	Macrophagocytic infiltration of the adenohypophysis	CD68(+)	TSS→TSS	Y	108
36	Our case	F/36	Pituitary mass with peripheral enhancement, PST	Tense cystic lesion with yellowish pus-like fluid	Inflammatory infiltration by foamy histiocytes and lymphocytes, with fibrosis, cyst and necrosis	CD68(+), CD1a(-), S-100(-)	TSS+HRT	N	6

MRI, magnetic resonance imaging; IHC, immunohistochemistry; F, female; M, male; NA, not available; PST, pituitary stalk thickening; TSS, transsphenoidal surgery; TCS, transcranial surgery; HRT, hormone replacement therapy; RT, radiation therapy; N, no; Y, yes.

Data on anterior pituitary function before surgery were available in 33 cases (**Figure 3B**). Hypogonadotropic hypogonadism, adrenocortical insufficiency, central hypothyroidism and growth hormone (GH) deficiency occurred in 63.6%, 54.6%, 54.6% and 36.4% of patients, respectively. In addition, 42.4% of patients had hyperprolactinemia.

The MRI presentation of patients with XHP is shown in **Table 2**. Based on the available data, the MRI scan showed a cystic mass with ring or peripheral enhancement in almost all of these patients. The mean maximum diameter of these lesions was 16.3 ± 4.8 mm (data available in 20 patients). In addition, pituitary stalk thickening (PST) was shown in 50.0% of patients.

A total of 35 patients underwent surgery, including one who received craniotomy and 34 (97.14%) who received transsphenoidal surgery (**Figure 3C**). Based on the available data, total lesion resection was achieved in 57.14% of patients (**Figure 3D**). New-onset transient DI was recorded in 3 patients, and rhinorrhea appeared in one patient. Follow-up data were available in 17 patients. The median follow-up time was 8.0 (4.5, 16.5) months, ranging from 2.0 to 108.0 months. Notably, the recurrence rate of patients who underwent partial lesion resection and biopsy was significantly higher than that of patients who received total resection (57.1% *vs.* 0.0%, *P* = 0.0147) (**Figure 3E**). Of the four patients who developed recurrence after surgery, one received high-dose glucocorticoid therapy, and the mass showed reduction three months later (12); one patient suffered recurrence again after repeat surgery, and radiation therapy was given (14); one patient was treated with high-dose steroids, but the prognosis was unknown (13); and one patient underwent reoperation and achieved total tumor resection with no recurrence during the 9-year follow-up (22).

The most common pituitary hormone abnormality to recover is hyperprolactinemia (100.0%), followed by GH deficiency (91.7%), hypogonadism (71.4%) and hypothyroidism (61.1%) postoperatively. Recovery of adrenocortical insufficiency was only noted in 38.9% of patients. In addition, 53.8% of patients with CDI were cured through surgery (**Figure 3B**).

The pathological results were available for 30 cases. The descriptions of “foamy histocytes”, “lymphocytes”, “fibrosis”, “hemosiderin” and “necrosis” appeared in 20 (66.7%), 17 (56.7%), 8 (26.7%), 4 (13.3%), 4 (13.3%), and 3 (10.0%) cases, respectively. The immunohistochemical results were available for 26 cases. CD68 status results were reported in 19 cases, and all showed positivity. Of the 11 cases reporting CD1a results and 12 cases reporting S-100 results, 100.0% and 91.7% showed negativity, respectively (**Table 2**).

## Discussion

Based on the histopathologic features, primary hypophysitis can be divided into five categories: lymphocytic hypophysitis (LHP), granulomatous hypophysitis (GHP), IgG-4 hypophysitis (IgG4HP), XH and necrotizing hypophysitis (NHP) (1, 23). The first two are main forms, and the last three are rare variants (1). As the most common form of hypophysitis, LHP predominantly occurs in females, especially during pregnancy (2, 24). Approximately 20-50% of cases with LHP have been shown to have other autoimmune diseases (25). Similar to patients with XHP, headaches (48.0%) and visual defects (40.0%) are the most frequent complaints in patients with LHP, and 70.0% have anterior pituitary dysfunction (26). In addition, CDI occurs in up to 70.0% of cases with LHP (26) but is relatively uncommon (36.1%) in XHP. GHP is the second most common subtype of primary hypophysitis and shows a female predilection (23, 27). Headaches and visual changes present in 61.0% and 40.2% of cases with GHP, but CDI only occurs in 26.8% of these patients (27). Unlike other subtypes of primary hypophysitis, IgG4HP is usually found in the context of IgG4-related disease and mainly occurs in males and older patients (mean age: 64.2 years) (23, 28). A total of 52.4%, 26.2% and 17.9% of patients with IgG4HP have panhypopituitarism, anterior hypopituitarism and CDI, respectively (28). In addition, NHP is the rarest subtype of primary hypophysitis and is thought to be a severe form of one of the other subtypes of hypophysitis (23). Because XHP has similar clinical presentations to other subtypes of hypophysitis, it is difficult to differentiate these disease types by symptoms alone.

Interestingly, five patients with XHP had other autoimmune diseases. Thus, there are some views that an autoimmune phenomenon may participate in the pathogenesis, and XHP is thought to be part of a systemic autoimmune condition (12, 13). In addition, XHP may develop as a local reaction to hemorrhage, necrosis and inflammation, but the pathogenesis remains unclear (13, 23).

MRI is an important tool in the diagnosis of pituitary diseases that can provide some clues for differential diagnosis. A finding in our study was that PST occurred in half of cases with GHP. PST can be seen in a variety of diseases (29, 30). A retrospective study that summarized 321 patients with PST in our center, showed that the etiologies of PST included neoplastic (75.2%), inflammatory (13.1%), and congenital diseases (11.7%) (30). It is not difficult to identify benign tumors such as craniopharyngioma, pituitary adenoma and meningioma and Rathke cleft cyst by MRI because these diseases usually have typical imaging features (31). And age, medical history, and laboratory examination help identify germ cell tumors and metastatic tumors (32). Besides, histiocytosis such as Langerhans cell histiocytosis (LCH) and Erdheim-Chester disease (ECD) can also affect the pituitary stalk, and diabetes insipidus is found in a quarter of patients with LCH and ECD (33, 34). However, both LCH and ECD can affect multiple systems (33, 34) and long-bone involvement is considered to be a hallmark of ECD (34). Therefore, multi-organ radiological screening and appropriate histology are sufficient for differential diagnosis (33, 34).

In our study, we found that most XHPs manifest as cystic sellar lesions with peripheral enhancement on MRI. However, this performance is also seen in both LHP and NHP (24). Thus, the differential diagnosis of hypophysitis by radiological examination is still a challenge. In this situation, biopsy seems to be unavoidable to make a definitive diagnosis. As one of the largest pituitary centers in China, we have performed hundreds of biopsy procedures on patients with PST or CDI in the last few years (30, 32). According to our experience, transsphenoidal biopsy is a safe and effective way to diagnose these indistinguishable sellar lesions (32). Therefore, for sellar lesions that are difficult to identify by clinical manifestations, laboratory examinations and radiological features, biopsy surgery should be considered, especially in experienced pituitary centers. According to previous descriptions in the literature, the typical gross presentation of XHP is a cystic lesion with yellowish pus-like fluid (**Table 2**). In addition, based on our intraoperative findings, the lesion had a thick wall, and the texture was tough.

The common feature of primary hypophysitis is inflammatory infiltration of lymphocytes and plasma cells into the pituitary gland, but the individual features among different subtypes differ except LHP (1). The diagnosis of GHP can be confirmed by the presence of multinucleated giant cells, and IgG4HP is characterized by IgG4-positive plasma cells (1, 2, 23). The typical pathological feature of XHP is foamy histiocyte infiltration, which can be marked by CD68 glycoprotein on immunohistochemistry (**Table 2**). Since Langerhans cells stain positive for CD1a, S-100 and CD207 (33), the absence of CD1a and S-100 helps to rule out LCH. Thus, histopathology remains the gold-standard diagnostic criterion for different forms of primary hypophysitis and histiocytosis.

Unlike LHP, which usually presents as a self-resolving process and has a good response to glucocorticoid therapy (35), clinical experience in the treatment of XHP is extremely limited. Although Joung et al. (12) reported a patient with recurrent XHP whose mass decreased after high-dose glucocorticoid therapy, and another patient showed less response to steroid therapy (14). In addition, tentative bromocriptine therapy failed to reduce the volume of the mass (5). Our study found that the recurrence rate was significantly lower in patients who underwent total lesion resection than in those who underwent partial resection or biopsy. In addition to the adrenocortical axis, anterior pituitary function can improve obviously, and more than half of patients with CDI can be cured through surgery. Therefore, surgical resection should be considered, especially for patients suffering from mass effects and pituitary dysfunction, and total mass resection is recommended. In addition, close follow-up and routine assessment of pituitary function are essential, and hormone replacement therapy should be given postoperatively when necessary (2, 23). Nevertheless, there were still two problems in the treatment of XHP. On the one hand, the lack of follow-up data does not allow for an accurate assessment of the long-term recurrence of XHP after surgery, resulting in a lack of best evidence for the treatment. On the other hand, the gross appearance of XHP—as well as the tough texture of the mass— is unfamiliar to surgeons, which leads some patients to undergo only partial resection or biopsy. Therefore, we have summarized the characteristics of XHP to increase surgeons’ awareness of this rare disease.

There were some limitations in our study. First, due to the rarity of XHP, selection bias was intrinsic to this investigation. Second, the majority of data were extracted from descriptions in previous literature, and this may have influenced the accuracy and integrity of the results. Third, due to the heterogeneity of surgical techniques in different centers and the incompleteness of follow-up data, the optimal treatment and long-term prognosis for XHP is still unclear. Therefore, the establishment of a database of rare diseases and close follow-up should be emphasized to provide evidence for guiding the diagnosis and treatment of these rare diseases.

## Conclusion

XHP is an extremely rare form of primary hypophysitis. Due to the similarity of its clinical manifestations and radiological findings to those of other forms of primary hypophysitis, the diagnosis and differential diagnosis of XHP are difficult to reach without a pathological examination. According to our study, pituitary dysfunction can be improved through surgery, and patients who undergo total lesion resection have a relatively low recurrence rate. Thus, surgery is both a diagnostic and a therapeutic method. However, because of the limited data, the real prognosis of this rare disease is still unclear.

## Data Availability Statement

The original contributions presented in the study are included in the article/supplementary material. Further inquiries can be directed to the corresponding authors.

## Author Contributions

YY, HZ, and JZ: conception and design. JZ, ZW, and YZ: development of methodology. JZ, ZW, XL, JL, and SJ: acquisition and analysis of data. JZ, ZW, and YY: writing, review, and revision of manuscript. WW and JF: technical and material support. YY, HZ, LD, and KD: study supervision. All authors have read and approved the manuscript. All authors contributed to the article and approved the submitted version.

## Funding

This study was supported by the Chinese Academy of Medical Sciences Innovation Fund (CAMS-2016-I2M-1-002).

## Conflict of Interest

The authors declare that the research was conducted in the absence of any commercial or financial relationships that could be construed as a potential conflict of interest.

## Publisher’s Note

All claims expressed in this article are solely those of the authors and do not necessarily represent those of their affiliated organizations, or those of the publisher, the editors and the reviewers. Any product that may be evaluated in this article, or claim that may be made by its manufacturer, is not guaranteed or endorsed by the publisher.
