# Corrigendum: Xanthomatous Hypophysitis: A Case Report and Comprehensive Literature Review

**DOI:** 10.3389/fendo.2021.797828

**Published:** 2021-11-09

**Authors:** Jianyu Zhu, Zhicheng Wang, Wenze Wang, Jinghua Fan, Yi Zhang, Xiaoxu Li, Jie Liu, Shenzhong Jiang, Kan Deng, Lian Duan, Yong Yao, Huijuan Zhu

**Affiliations:** ^1^ Department of Neurosurgery, Peking Union Medical College Hospital, Chinese Academy of Medical Science and Peking Union Medical College, Beijing, China; ^2^ Department of Neurosurgery, The First Affiliated Hospital of Fujian Medical University, Fuzhou, China; ^3^ Department of Pathology, Peking Union Medical College Hospital, Chinese Academy of Medical Science and Peking Union Medical College, Beijing, China; ^4^ Key Laboratory of Endocrinology of National Health Commission, Department of Endocrinology, Peking Union Medical College Hospital, Chinese Academy of Medical Science and Peking Union Medical College, Beijing, China

**Keywords:** xanthomatous hypophysitis, clinical characteristics, pathological examination, surgery, recurrence

In the published article, there was an error regarding the affiliations for author Jianyu Zhu. As well as having affiliation 2, he should also be assigned affiliation 1:

Department of Neurosurgery, Peking Union Medical College Hospital, Chinese Academy of Medical Science and Peking Union Medical College, Beijing, China

The authors apologize for this error and state that this does not change the scientific conclusions of the article in any way. The original article has been updated.

## Publisher’s Note

All claims expressed in this article are solely those of the authors and do not necessarily represent those of their affiliated organizations, or those of the publisher, the editors and the reviewers. Any product that may be evaluated in this article, or claim that may be made by its manufacturer, is not guaranteed or endorsed by the publisher.

